# Axillary artery compromise in a minimally displaced proximal humerus fracture: a case report

**DOI:** 10.1186/1757-1626-2-9308

**Published:** 2009-12-11

**Authors:** Mohamed Sukeik, Girish Vashista, Nebal Shaath

**Affiliations:** 1 Department of Trauma and Orthopaedics, Barnsley District General Hospital, Hillder House, 49-51 Gawber Road, Barnsley, S75 2PY, UK

## Abstract

Minimally displaced fractures of the surgical neck of the humerus are rarely associated with axillary artery injury. The innocuous appearance of the x-rays can be misleading and a missed arterial injury in these fractures could potentially lead to disastrous consequences. We report the case of a patient who sustained a minimally displaced fracture of the proximal humerus with vascular compromise requiring immediate investigation and referral to vascular surgeons. Despite spontaneous resolution of the vascular insult, it is important to remember the association of such fractures with vascular injuries in order to diagnose them early and prevent serious complications including amputation.

## Introduction

Minimally displaced fractures of the neck of the humerus are rarely associated with injury to the axillary artery [1]. In this context, the innocuous appearance of the x-rays can mislead the treating doctor into a state of complacency unless a thorough clinical examination is carried out in the Emergency Department. A missed arterial injury in these fractures, though infrequent, could potentially lead to disastrous consequences.

A relevant case is discussed and mechanisms related to vascular injuries in association with proximal humerus fractures are described with the emphasis on having a low threshold of suspecting and immediately treating such injuries in order to prevent catastrophic results.

## Case presentation

**Figure 1:**
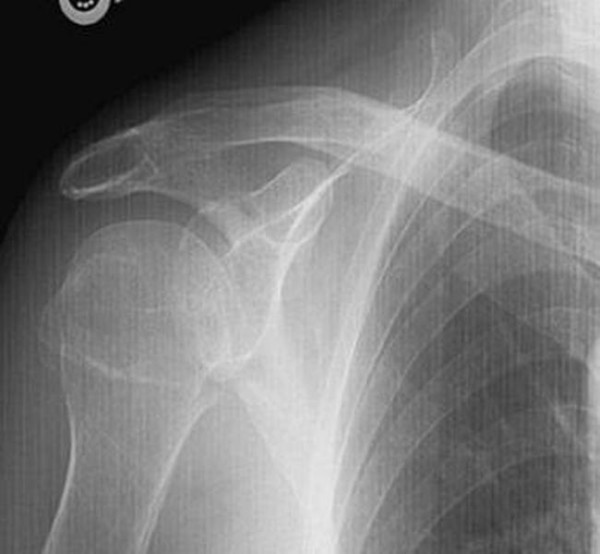
**AP view showing minimally displaced fracture of proximal humerus**.

**Figure 2:**
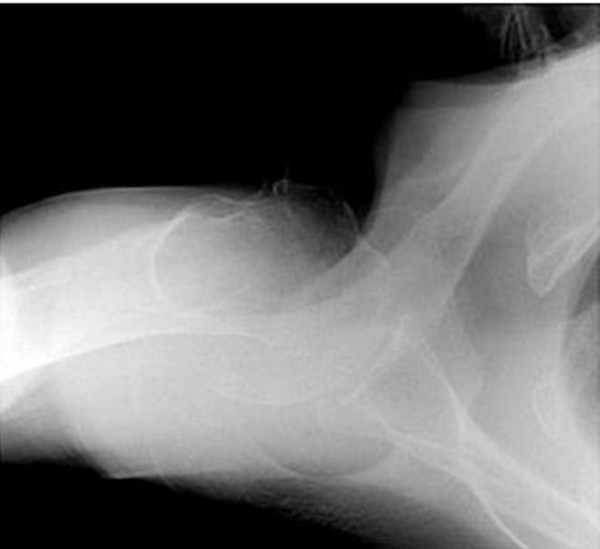
**Axial view**.

**Figure 3:**
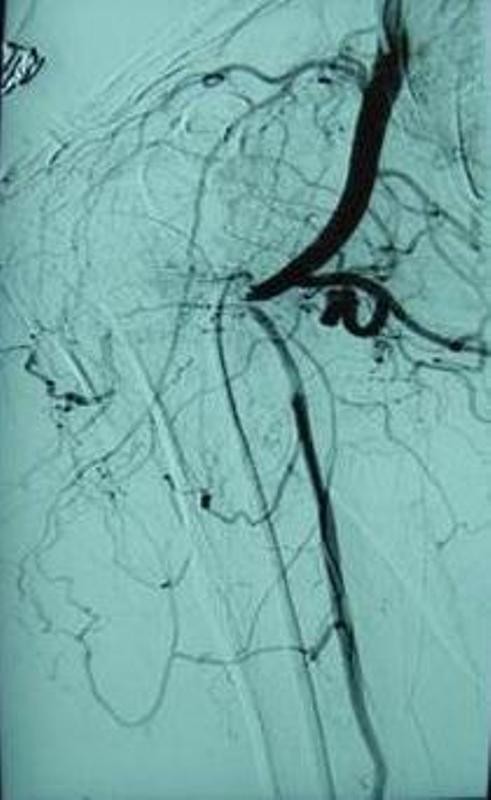
**Angiography showing obstruction to flow at neck of humerus**.

**Figure 4:**
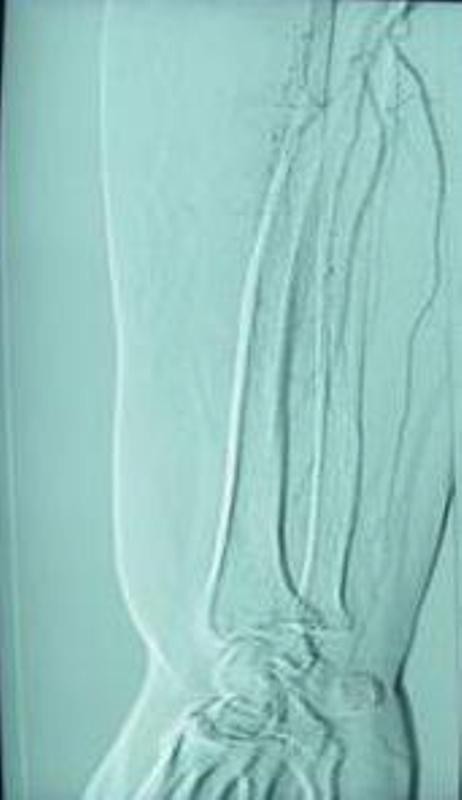
**Diminished flow in radial and ulnar arteries**.

A 74-year-old white British lady attended the Accident and Emergency department after having fallen from a low height at home. She complained of pain around the right shoulder and had bruising extending from the shoulder to the elbow. There was no gross deformity of the shoulder. The fingers were cold to the touch with a delayed capillary refill. The nail beds were cyanosed. Further examination of the right upper limb revealed loss of pulsations in the brachial and radial arteries with preservation of sensations in the hand. A Doppler ultrasound also failed to detect distal pulsations. X-rays of the shoulder showed a minimally displaced fracture of the surgical neck of the humerus (Figure 1, Figure 2). Vascular surgeons were consulted immediately and an angiogram performed showed kinking and compression of the axillary artery at the fracture site (Figure 3) and diminished blood flow distal to the axillary artery (Figure 4). However, the patient spontaneously recovered her pulse before any orthopaedic intervention was made; it was deemed unnecessary to fix the fracture because of its minimally displaced nature. The vascular surgeons also decided to treat the arterial injury conservatively.

## Results

The patient was admitted for monitoring of the distal pulses, but no further loss of pulsations was documented. At 12 months of follow-up, she had good functional outcome with a normally perfused limb.

## Discussion

Fractures of the proximal humerus account for 4-5% of all fractures [2]. Axillary artery injury in association with proximal humeral fractures remains low with respect to the close anteromedial relationship of the artery to the proximal humerus. The incidence of brachial plexus injury is relatively more common in these fractures [3]. Vascular injuries associated with proximal humerus fractures are more common in elderly patients [4]. The combination of osteoporosis and atherosclerosis may take part in the pathogenesis of this injury. Several authors have reported axillary artery injury following displaced fractures of the proximal humerus with or without subluxation [5,6]. However, such injuries are rarely associated with minimally displaced proximal humerus fractures.

There are several mechanisms by which the axillary artery can be injured in proximal humeral fractures. A direct injury to the artery by a sharp bony fragment can cause laceration and rupture [1], violent overstretching can result in rupture especially in an atheromatous artery [6] and another important mechanism of injury is intimal tear and thrombosis. The artery stretches across the bony fragment, the adventitia remains intact and the fragile intima tears, leading to thrombosis. In our case, the angiography showed kinking and compression of the axillary artery at the fracture site which probably resolved spontaneously prior to intimal injury and thrombus formation. Axillary artery injury has also been described in a case of blunt injury to the shoulder without any fractures. Angiography in that case showed leaking of contrast material from a branch of the axillary artery. At formal exploration, a side branch of the axillary artery was found to be avulsed from the artery itself [7].

Diagnosis of axillary artery injury may be difficult as peripheral pulses may remain intact initially and later disappear. As a result, vascular injury can occasionally manifest several days after a fracture of the proximal humerus [8].

Paraesthesia is probably the most reliable symptom of inadequate distal circulation and should always be taken seriously. Collateral circulation around the shoulder is effective, and depending on the level of injury to the axillary artery, distal circulation might remain adequate and the patient asymptomatic [1]. In Drapanas's series, distal pulsations were present in 27% of the patients with major arterial injury [9]. Amputation rates following axillary artery ligation have been reported to be as high as 43% [10]. However, true arterial spasm occurs extremely rarely and is a presumptive, dangerous diagnosis to make [11].

## Conclusion

Arterial injuries associated with proximal humeral fractures can be easily missed. Our case illustrates that minimally displaced fractures of the surgical neck of the humerus can be associated with vascular compromise and this can occur in the elderly with lesser degrees of force because of atherosclerotic rigidity of the artery. Potential complications can be avoided by careful examination of the patient and avoiding treatment of the x-ray alone.

Once a vascular injury is suspected, Doppler examination is necessary to establish the magnitude and quality of the arterial signal. Angiography should be performed immediately if arterial compromise is suspected after Doppler examination, and vascular surgeons should be consulted early in the management of such patients.

## Consent

Written informed consent was obtained from the patient for publication of this case report and accompanying images. A copy of the written consent is available for review by the Editor-in-Chief of this journal.

## Competing interests

The authors declare that they have no competing interests.

## Authors' contributions

MS co-ordinated the entire effort and wrote the manuscript. GV helped with the draft of the case report, and obtained consent from the patient. NS was the treating and supervising consultant in charge. All authors read and approved the final manuscript.

## References

[B1] Hayes JM Van Winkle G Axillary artery injury with minimally displaced fracture of the neck of the humerus J Trauma 1983 23 5 431 433 10.1097/00005373-198305000-00014 6854682

[B2] Neer CS Rockwood CA Jr, Green DP Fracture and Dislocations of the Shoulder Fractures 1975 1 2 Philadelphia: Lippincott 593 594

[B3] Yagubyan M Panneton JM Axillary artery injury from humeral neck fracture: A rare but disabling traumatic event Vascular and Endovascular Surgery 2004 38 2 175 185 10.1177/153857440403800210 15064849

[B4] Neer CS Displaced proximal humerus fractures. Part II: Treatment of three-part and four-part displacement JBJS 1970 52A 1077

[B5] Rich NM Spencer FC Vascular Trauma 1978 Philadelphia: WB Saunders 330 347

[B6] Theodorides T deKeizer G Injuries of the axillary artery caused by fractures of the neck of the humerus Injury 1976 8 2 120 123 10.1016/0020-1383(76)90045-0 1002286

[B7] MacNamara AF Ismail A Combined brachial plexus and vascular injury in the absence of bony injury J Accid Emerg Med 2000 17 378 379 10.1136/emj.17.5.378 PMC1725456 11005418

[B8] Smyth EHJ Major arterial injury in closed fracture of the neck of the humerus: report of a case J Bone Joint Surg Br 1969 51 3 508 510 5820797

[B9] Jensen BV Jacobsen J Andreasen H Late appearance of arterial injury caused by fracture of the neck of the humerus J Trauma 1987 12 1368 1369 10.1097/00005373-198712000-00010 3694729

[B10] Laverick MD Barros AAB Kirk SJ Mollan RAB Management of blunt injuries of the axillary artery and the neck of the humerus: case report J Trauma 1990 30 3 360 361 10.1097/00005373-199003000-00022 2313762

[B11] Rob CG Standeven A Closed traumatic lesions of the axillary and brachial arteries Lancet 1956 1 597 599 10.1016/s0140-6736(56)90645-6 13320815

